# Health Information-Seeking Behaviour on High-Risk Behaviour among Adolescents

**DOI:** 10.21315/mjms2023.30.5.15

**Published:** 2023-10-30

**Authors:** Nabila Iezzah Ab Hakimee, Ashikin Atan, Sutantri Sutantri, Siew Pien Lee

**Affiliations:** 1 National Heart Institute, Kuala Lumpur, Malaysia; 2 Department of Professional Nursing Studies, Kulliyyah of Nursing, International Islamic University Malaysia, Pahang, Malaysia; 3 School of Nursing, Faculty of Medicine and Health Sciences, Universitas Muhammadiyah Yogyakarta, Yogyakarta, Indonesia; 4 Department of Special Care Nursing, Kulliyyah of Nursing, International Islamic University Malaysia, Pahang, Malaysia

**Keywords:** health information, information seeking behaviour, health information-seeking behaviour, high-risk behaviour, adolescents

## Abstract

**Background:**

The unique nature of adolescence makes youths highly susceptible to high-risk behaviours. Thus, prevention and health promotion are imperative for this influential age. Despite various approaches towards health promotion, knowledge related to adolescent health is still low among Malaysian adolescents. This study aims to investigate adolescent health information-seeking behaviours related to high-risk behaviours.

**Methods:**

A cross-sectional study was conducted among 370 adolescents aged 10 years old–19 years old throughout Malaysia. The questionnaire used was adapted from a previous study and the pilot study resulted in Cronbach’s alpha of 0.85. IBM SPSS Statistics version 25.0 software was used for data analysis at two statistical levels: descriptive and inferential (Mann-Whitney U test).

**Result:**

The most important health information needs related to high-risk behaviour according to the adolescents were ‘violence’ (3.72 score out of 5), ‘sexual activity-related disease’ (3.64 score out of 5) and ‘physical activity and effect of lack in physical activity’ (3.61 score out of 5). ‘Physician’ (4.01 score out of 5) and ‘the internet’ (3.95 score out of 5) were the most important sources for obtaining health information related to high-risk behaviours. The main criterion for the quality of health information was the ‘validity and reliability of the information’ (4.55 score out of 5). The findings indicate that adolescents have a positive attitude towards health information-seeking behaviour, although slight differences between boys and girls are exhibited. The most common barrier to health information seeking experienced by adolescents is ‘difficulty in determining the quality of information found’.

**Conclusion:**

Adolescents tend to use professional and informal sources, have good criteria in the selection of information and have a considerably high interest in seeking health information related to high-risk behaviour.

## Introduction

There are approximately 1.3 billion of adolescents aged 10 years old–19 years old worldwide, making up 16% of the world population (1) and there were an estimated 5.4 million adolescents in Malaysia in 2023 (2). Adolescents are the most valuable asset in the country, as they will become the leaders who will continue to sustain our national goals. Nonetheless, adolescence is a phase by which dramatic physical, sexual, psychological and social developmental changes occur simultaneously as the shift from childhood to adulthood takes place. Thus, they are particularly susceptible to high-risk behaviours, including self-injury, violence and suicide (e.g. driving-related risks, fighting and aggression), substance use (e.g. consumption of cigarettes, alcohol and drugs), obesity and unhealthy dieting, as well as risky sexual behaviours (3). These behaviours have adverse effects on overall development and well. Moreover, it is reported that over 1.5 million adolescents and young adults died in 2021 due to preventable causes, which include their high-risk behaviours (4). Hence, despite being considered the healthiest compared to other age groups, adolescents require unique health provisions which mainly focus on preventing high-risk behaviours.

If the quality and attributes of the individual in this age period are not guided correctly, the changes during this period of growth can cause problems, such as high-risk behaviours (5). Awareness of information about high-risk behaviours is one of the reasons adolescents get involved in information-seeking behaviours regarding health. The search for health information shall be called ‘health information-seeking behaviour’, as it is done for health purposes and is also a part of the decision-making process to implement health behaviour among adolescents (6).

In many developing countries, including Malaysia, there is a lack of tools or information to help adolescents make good health decisions (7). Many of the existing health information provided, especially for adolescents, concerns sexual health. The government has also conducted many plans and approaches towards health promotion. However, awareness of adolescent health is still low among Malaysian adolescents (2). This indicates the need to improve the promotion of related health or the nature of the health information provided. Hence, more research needs to be done so that health promotion to adolescents is simultaneously effective and supportive of adolescent health information-seeking behaviour.

A systematic review of the issue and trends in health information-seeking behaviours from the Malaysian perspective showed that a limited number of studies have been conducted, especially among adolescents (8). There have not been many studies on the behaviour of Generation Z which include individuals born between 1997 and 2012 in the context of seeking health information. To fill this gap, this study was designed to determine adolescent health information-seeking behaviour related to high-risk behaviour.

## Methods

### Study Setting and Design

A cross-sectional quantitative study was conducted among Malaysian adolescents through an online survey. Due to limitations caused by the COVID-19 pandemic, convenience sampling was used with the distribution of online surveys. Calculated using Raosoft sample size calculator (9), the recommended sample size was 285 with a confidence level of 95% and a margin error of 5%. The data collection was carried out from October 2022 to December 2022. Within the stipulated time, 370 students answered the survey and were involved in the study.

### Materials

The questionnaire for the survey was adapted from a previous study that achieved a Cronbach’s alpha of 0.85 (10). It was translated from English to Malay and back translation was done. The Cronbach’s alpha results of a pilot study among 40 participants using the questionnaire, which included both languages, ranged from 0.64 to 0.89. The questionnaire consisted of the following items: demographic characteristics, types of medical and health information needed, sources of medical and health information, criteria for the quality of the information and barriers to accessing medical and health information.

### Data Collection

The data was collected from November 2020 to January 2021. Adolescents who met the inclusion criteria (aged from 10 years old to 19 years old and had obtained parental consent) were invited to answer an online questionnaire that was disseminated through a few electronic or online platforms, including Facebook and WhatsApp, via a link. Adolescents who did not fulfil the inclusion criteria were excluded from the study.

An information sheet was attached to the questionnaire, and the contents consisted of an explanation of the purpose of the study, procedure, confidentiality and the right to withdraw, as well as the contact information of the researcher. All the information given by the participants was kept confidential throughout the study process. The analysis was done using IBM SPSS Statistics version 25.0.

## Results

### Sociodemographic Data

Table 1 presents the sociodemographic characteristics data of the participants in this study. In terms of gender, 273 of the total respondents were female (73.8%), and the remaining 97 respondents (26.2%) were male. The adolescent age was divided into three categories: i) early adolescence (10 years old–14 years old), middle adolescence (15 years old–17 years old) and late adolescence (18 years old–19 years old). Among the adolescents, 84 (22.8%) were in early adolescence, 181 (49%) were in middle adolescence and 105 (28.4%) were in late adolescence. For the level of education, 24 (6.5%) respondents were currently studying in primary school (Standard 4, Standard 5 and Standard 6), 245 (66.3%) in middle school (Forms 1–5) and 101 (27.3%) in Form 6, diploma or matriculation. A total of 210 (56.8%) respondents had a family income of below RM4,850 (B40) and the rest (43.2%) were among the M40 and T20 families, which had a family income of more than RM4,850. In terms of ethnicity, most of the respondents were Malay, which contributed 97.6% of the total number of respondents.

### Adolescent Health Information-Seeking Behaviour Related to High-Risk Behaviour

Table 2 shows the findings regarding health information related to the high-risk behaviours that were needed and sought by the adolescents. The most popular health information needs related to high-risk behaviour according to the adolescents were ‘violence’ (3.72 score out of 5), ‘sexual activity-related disease’ (3.64 score out of 5) and ‘physical activity and effect of lack in physical activity’ (3.61 score out of 5). However, other information including ‘tobacco abuse’ (3.48 score out of 5), ‘incidents and injuries’ (3.46 score out of 5), and ‘narcotics and alcohol’ (3.41 score out of 5) also had considerably high mean scores; hence, all these topics were important to the adolescents.

From Table 3, the most important sources to obtain health information related to high-risk behaviours among the studied adolescents were ‘physician or other members of healthcare’ (4.01 score out of 5), ‘the internet’ (3.95 score out of 5), ‘family members’ (3.81 score out of 5), TV (3.77 score out of 5) and ‘workshops’ (3.72 score out of 5) and the least important source to obtain health information related to high-risk behaviours was from ‘radio’ with a mean score of 3.14 out of 5.

Table 4 presents the research findings regarding the criteria for the selection of health information sources that showed ‘higher reliability in terms of information’ and ‘ease of access’ (49.7% and 30.8%, respectively) were the most important criteria for the selection of health information sources related to high-risk behaviours. In total, 12.7% of the adolescents preferred confidentiality of information and 5.9% preferred lower costs when selecting health information. Other preferences, including ‘capability of the staff’, ‘self-consciousness’ and ‘passion’, also affect their selection of health information.

Table 5 reports that among the 370 adolescents who had completed the questionnaire, 352 (95.1%) used ‘the internet’ to obtain health information related to high-risk behaviours during the past month, 40.8% chose ‘search engines’, 23.2% chose the ‘website of healthcare centres’ and 17.6% chose ‘specific website in the health domain’ as places where they usually start their search on the internet.

From Table 6, the most important criteria for the quality of health information related to high-risk behaviours among the participants were ‘validity and reliability of the information’ (4.55 score out of 5), ‘the trueness and correctness of the information’ (4.53 score out of 5), ‘understandability of the information content’ (4.34 score out of 5), ‘keeping the information up to date’ (4.32 score out of 5) and ‘the availability of the author’s postal address and phone number’ (3.48 score out of 5).

### Gender and Health Information-Seeking Behaviour Among Adolescents

Table 7 shows that the findings using the Mann-Whitney U test indicated a significant difference between gender and the need for information about ‘incident and injuries’ and ‘narcotics and alcohol’. The need for information about ‘incident and injuries’ is higher among girls than boys, while the need for information on ‘narcotics and alcohol’ is higher among boys than girls.

Table 8 shows that there are no significant differences between boys and girls when it comes to preferred sources of health information, using the Mann-Whitney U test.

Based on the Mann-Whitney U test, Table 9 indicates that the median of information quality sought by adolescents between boys and girls is the same except for the criteria of ‘free access to information’. Boys are more likely to look for ‘free access to information’ in searching for health information as compared to girls. The difference between boys and girls in this criterion is significant in terms of the median score (4.26 for boys and 4.01 for girls).

### Barriers to Health Information-Seeking Related to High-Risk Behaviour

Table 10 presents the most difficult points and barriers to adolescents accessing and using health information related to high-risk behaviours, which were ‘difficulty in determining the quality of information found’ (3.40 score out of 5) and ‘concerns about the disclosure of their problems or illness to others’ (3.28 score out of 5), with ‘being punished by their parents or school officials’ (2.13 out of 5) being the least important.

## Discussion

### Type of Medical and Health Information Related to High-Risk Behaviour Needed by Adolescents

The results showed that the most important health information related to adolescents’ high-risk behaviour needed by adolescents was on ‘violence’, ‘sexual activity-related disease’ and ‘physical activity and effect of lack in physical activity’. However, other types of medical information, which include information on ‘tobacco abuse’, ‘incidents and injuries’ and ‘narcotics and alcohol’, were also important to the adolescents, as they also showed considerably high interest in seeking information on these topics. This finding is consistent with another study that was conducted on high-risk behaviour among adolescents in Malaysia (11) and found that the most common high-risk behaviour among Malaysian adolescents was physical inactivity, followed by smoking, and the least common high-risk behaviour among adolescents was alcohol consumption (11). The findings were also consistent with another study among adolescents in Iran, which showed that the highest needs for obtaining information were about ‘lack of mobility’, ‘high-risk sexual behaviours’ and ‘incidents and injuries’ (10).

Furthermore, the need for information about ‘incidents and injuries’ was found to be higher among girls than boys, while the need for information on ‘narcotics and alcohol’ was higher among boys than girls. Meanwhile, there were no significant differences observed between boys and girls in the need for information for other subjects, such as ‘tobacco abuse’, ‘physical activity and the effect of lack in physical activity’, ‘violence’ and ‘sexual activity-related disease’. The degree of need for information between girls and boys was inconsistent with the results from the study conducted in Iran, where they found that the needs for information on ‘incident and injuries’ and ‘physical violence’ were higher in boys than in girls (10).

### Sources of Health Information Related to Adolescents’ High-Risk Behaviours

The most important sources for obtaining health information related to high-risk behaviours among the studied adolescents are physicians or other health staff, the internet, family members, TV and workshops, while the least important is radio. Similarly, in the Iranian study, the main sources for obtaining health information related to high-risk behaviours among adolescents include the internet and family members (10). This pattern is also found in another study on how adolescents access health information, which stated that the majority of the adolescents were more comfortable using the internet as their source of health information related to high-risk behaviours (12). However, they also reported a contradictory finding: despite the fact that physicians are among the most accurate sources of health information, they were often underused by adolescents in their study (12).

The most preferred place to start the search for health information on the internet was search engines like Yahoo and Google, followed by specific websites in the health domain and websites of healthcare centres. This finding is consistent with previous studies, which found that search engines were the most chosen initial platform for searching for health information (10, 13). The criteria for the selection of health information sources showed that ‘higher reliability in terms of information’ and ‘ease of access’ were the main criteria that affected their selection of health information.

There are no significant differences in the sources of health information referred to by adolescent boys and girls. However, a previous study found gender differences in source preferences (14). In their study, boys were more likely to find the internet more convenient than girls. In the context of formal sources external to the school, girls are significantly likelier to find family planning clinics useful than boys (14).

### Criteria for the Quality of Information Sought by Adolescents

The most important criteria for the quality of health information related to high-risk behaviours were ‘validity and reliability of the information’, ‘the trueness and correctness of the information’, ‘understandability of the information content’ and ‘keeping the information up to date’, while the least important was ‘the availability of the author’s postal address and phone number’. Boys are more likely to look for information that allows free access when searching for quality health information than girls. Otherwise, no significant differences were found in other areas between girls and boys.

### Health Information-Seeking Attitudes Related to High-Risk Behaviour among Adolescents

The average score for the attitude towards medical and health information-seeking behaviour indicates that, overall, most adolescents have a positive attitude towards health information-seeking behaviour. Positive attitudes towards health information-seeking behaviour among adolescents were also seen in a previous study (10). Further findings showed that there were no significant differences in attitude scores between the genders.

### Barriers to Health Information-Seeking Behaviours among Adolescents

The most important difficult points and barriers to adolescents in accessing and using health information related to high-risk behaviours were ‘difficulty in determining the quality of information found’, ‘concerns about the disclosure of their problems or illness to others’ and ‘absence of appropriate information’, while the least important was ‘being punished by their parents or school officials’. These findings are consistent with the Iranian study, which concluded that the most rated problems for adolescents seeking health information were difficulty in identifying the quality of information, absence of suitable information, and concerns about the disclosure of problems or illness to others (10). Our study also shows that, generally, the barriers to health information seeking experienced by these adolescents are moderate. These findings are important as they highlight that adolescents need help seeking quality information. This can be due to the lack of exposure to the right techniques to search for reliable information on the web or in books. The lack of knowledge about the right way to search for health information is also related to the claim of the absence of appropriate information (15). This may also indicate that, regardless of the health information provided by the health domains, the information might not have reached adolescents effectively.

## Conclusion

This study elucidated the preferences and behaviours of adolescents in health information seeking, which is one of the necessary steps in developing effective health promotion and information. Improving adolescents’ level of health information knowledge can contribute to the development of effective preventive approaches, promotion of adolescents’ health and aid in modifying adolescents’ unhealthy behaviours. In summary, adolescents tend to use both professional sources and informal sources, have good criteria in the selection of information and have a considerably high interest in seeking health information related to high-risk behaviours. There are slight differences between the genders in health information-seeking behaviours among adolescents.

Based on the results obtained in this study, the most important health information needs according to the adolescents were violence, sexual activity-related diseases and physical inactivity. Physicians, the internet, family members, TV and workshops were the most important sources for obtaining health information related to high-risk behaviours. The adolescents’ criteria for the quality of health information related to high-risk behaviours were the validity and reliability of the information, the trueness and correctness of the information, clarity of the information content and keeping the information current. The barriers to health information seeking experienced by adolescents are moderate, with ‘difficulty in determining the quality of information found’ being the biggest barrier.

This study highlighted the need to provide valid health information through different information sources with regard to the level of skill and informational literacy, as positive attitudes were observed in the majority of adolescents. The official institutes, the Ministry of Health and the Ministry of Education, Malaysia can create more local health databases and simple reading materials proportionate to the age group of adolescents. Furthermore, the main barrier to health information seeking is the difficulty in determining the quality of information, indicating the necessity of providing training about health and media literacy. It is also recommended that specialists in health information domains consider evaluating and introducing valid websites in the field of health information to adolescents, especially on high-risk behaviours, to secure access to valid information.

## Figures and Tables

**Table 1:** Sociodemographic data of the study participant (*N* = 370)

Variables	Frequency (*n*)	%
Gender		
Male	97	26.2
Female	273	73.8
Age		
Early adolescents (10 years old–14 years old)	84	22.7
Middle adolescents (15 years old–17 years old)	181	48.9
Late adolescents (18 years old–19 years old)	105	28.4
Level of education		
Standard 4	1	.3
Standard 5	11	3.0
Standard 6	12	3.2
Form 1	43	11.6
Form 2	18	4.9
Form 3	33	8.9
Form 4	79	21.4
Form 5	72	19.5
Form 6/Matriculation/Diploma/Foundation	101	27.3
Family income		
B40 (< RM4,850)	210	56.8
Non-B40 (> RM4,850)	160	43.2
Races		
Malay	361	97.6
Chinese	7	1.9
India	1	0.3
Others	1	0.3
Religion		
Islam	362	97.8
Christian	1	0.3
Hindu	1	0.3
Buddha	6	1.6

**Table 2:** Mean scores of type of medical and health information related to high-risk behaviour and willingness to seek for (*N* = 370)

Type of medical and health information related to high-risk behaviour	Mean score out of 5	Rank
Incidents and injuries (accidents, falls from height, injuries, etc.)	3.46	5
Information on tobacco abuse (cigarettes, e-cigarette/vape, etc.)	3.48	4
Physical activity and the effect of lack in physical activity (sports, fitness, etc.)	3.61	3
Narcotics and alcohol (alcohol or drug rehabilitation)	3.41	6
Violence (physical violence)	3.72	1
Sexual activity-related disease (HIV, sexual diseases, etc.)	3.64	2

**Table 3:** The ranks and mean scores of health information sources related to adolescents’ high-risk behaviours

Sources for seeking medical and health information	Mean score out of 5	Rank
Physician or other members of the healthcare	4.01	1
TV	3.77	4
Radio	3.14	11
Friends or classmates	3.43	10
Internet	3.95	2
Virtual social media	3.68	6
Teachers and school officials	3.65	7
Mobile applications	3.65	8
Information sources available in public libraries	3.53	9
Family members	3.81	3
Workshops and meetings on health	3.72	5

**Table 4:** The criteria for the selection of health information

The criteria for the selection of health information	Frequency (%)
Ease of access	114 (30.8)
Higher reliability	184 (49.7)
Lower costs	22 (5.9)
Confidentiality of information	47 (12.7)
Others	3 (0.8)

**Table 5:** Frequency table for place to start the search for health information related to high-risk behaviour when using the internet

Place to start the search for health information related to high-risk behaviour when using the internet	Frequency (%)
i) Use the Internet to seek health information	352 (95.1)
ii) A search engine such as Yahoo, Google, etc	151 (40.8)
iii) A specific website in the health domain	65 (17.6)
iv) The website of a healthcare centre	86 (23.2)
v) Social media such as: Facebook, Telegram, WhatsApp, Instagram, etc	34 (9.2)
vi) Weblogs	4 (1.1)
vii) Electronic discussion groups	12 (3.2)

**Table 6:** The mean score and rank of the criteria for the quality of information

The criteria for the quality of information	Mean (score out of 5)	Rank
i) The expertise, experience, and reputation of the author of the content	4.17	8
ii) The availability of the author’s phone number and postal address	3.48	16
iii) The author’s dependence on a reputable and prestigious institute	3.74	15
iv) The simplicity of finding the information	4.07	10
v) Free access to information	4.08	9
vi) Provision of information about the terms and conditions of accessing (the observance of copyright)	3.96	12
vii) Providing the date of publishing the content	3.84	13
viii) Keeping the information up-to-date	4.32	4
ix) Impartiality and absence of bias (favouritism or supporting a certain person or organisation more than others)	4.22	6
x) The trueness and correctness of the information	4.53	2
xi) Validity and reliability of the information	4.55	1
xii) The breadth and scope of the information (the superficial or profound presentation of the content)	4.30	5
xiii) Understandability of the information content	4.34	3
xiv) Provision of new and innovative information	4.20	7
xv) Taking the audience into consideration (proportionally to age groups such as: school students, adults, etc.)	4.06	11
xvi) A friend’s recommendation to use a type of information; e.g. watching a certain satellite channel or joining a Telegram group	3.79	14

**Table 7:** The need for medical and health information related to high-risk behaviour (median) between male and female

Type of health information	Median (IQR)		*z*-statistic	*P*-value

	Male	Female		
i) Incident and injuries	3 (1.00)	4 (1.00)	−1.980	**0.048** *
ii) Information on tobacco abuse	4 (1.00)	3 (1.00)	−1.364	0.173
iii) Physical activity and the effect of lack in physical activity	4 (1.00)	4 (1.00)	−1.410	0.159
iv) Narcotics and alcohol	4 (1.00)	3 (1.00)	−2.312	**0.021** *
v) Violence	4 (1.00)	4 (1.00)	−0.090	0.928
vi) Sexual activity-related disease	4 (1.00)	4 (1.00)	−0.552	0.581

Note:

* *P* < 0.05, statistically significant

**Table 8:** The sources of health information related to high-risk behaviour (median) between male and female

Sources for seeking medical and health information	Median (IQR)		*z*-statistic	*P*-value

	Male	Female		
i) Physician or other members of the treatment staff	4 (1.00)	4 (1.00)	−0.242	0.809
ii) TV	4 (1.00)	4 (1.00)	−0.608	0.543
iii) Radio	3 (1.00)	3 (1.00)	−0.542	0.588
iv) Friends or classmates	4 (1.00)	3 (1.00)	−1.201	0.230
v) Internet	4 (1.00)	4 (1.00)	−0.149	0.882
vi) Virtual social media	4 (1.00)	4 (1.00)	−1.062	0.288
vii) Teachers and school officials	4 (1.00)	4 (1.00)	−1.438	0.150
viii) Mobile applications	4 (1.00)	4 (1.00)	−0.874	0.382
ix) Information sources available in public libraries	4 (1.00)	4 (1.00)	−0.294	0.769
x) Family members	4 (1.00)	4 (1.00)	−0.605	0.545
xi) Workshops and meetings on health	4 (1.00)	4 (1.00)	−0.396	0.692

**Table 9:** The criteria for the quality of information sought by adolescents (median) between male and female

The criteria for the quality of information	Median (IQR)		*z*-statistic	*P*-value

	Male	Female		
i) The expertise, experience, and reputation of the author of the content	4 (1.00)	4 (1.00)	−1.473	0.141
ii) The availability of the author’s phone number and postal address	4 (1.00)	4 (1.00)	−0.115	0.909
iii) The author’s dependence on a reputable and prestigious institute	4 (1.00)	4 (1.00)	−1.610	0.108
iv) The simplicity of finding the information	4 (1.00)	4 (1.00)	−1.205	0.228
v) Free access to information	4 (1.00)	4 (1.00)	−2.226	**0.026** *
vi) Provision of information about the terms and conditions of accessing (the observance of copyright)	4 (1.00)	4 (1.00)	−0.349	0.727
vii) Providing the date of publishing the content	4 (1.00)	4 (1.00)	−0.898	0.369
viii) Keeping the information up-to-date	4 (1.00)	4 (1.00)	−0.802	0.422
ix) Impartiality and absence of bias (favouritism or supporting a certain person or organisation more than others)	4 (1.00)	4 (1.00)	−0.039	0.969
x) The trueness and correctness of the information	5 (1.00)	5 (1.00)	−1.305	0.192
xi) Validity and reliability of the information	5 (1.00)	5 (1.00)	−0.863	0.388
xii) The breadth and scope of the information (the superficial or profound presentation of the content)	4 (1.00)	4 (1.00)	−0.169	0.866
xiii) Understandability of the information content	4 (1.00)	4 (1.00)	−0.907	0.364
xiv) Provision of new and innovative information	4 (1.00)	4 (1.00)	−0.350	0.726
xv) Taking the audience into consideration (proportionally to age groups such as: school students, adults, etc.)	4 (1.00)	4 (1.00)	−0.119	0.905
xvi) A friend’s recommendation to use a type of information; e.g. watching a certain satellite channel or joining a Telegram group	4 (1.00)	4 (1.00)	−0.551	0.582

Note:

* *P* < 0.05, statistically significant

**Table 10:** Barriers toward health information seeking related to high-risk behaviour

Barriers toward health information seeking	Mean	Rank
i) Lack of access to appropriate and practical information sources in a simple language	3.08	4
ii) Concerns about the disclosure of their problems or illness to others	3.28	2
iii) High costs of access to medical and health information	2.97	5
iv) Believing that they can solve the problem or the disease themselves.	2.54	7
v) Being punished by their parents or school officials	2.13	8
vi) Lack of information or inability to find the information being searched for	2.91	6
vii) Difficulty in determining the quality of information found	3.40	1
viii) The absence of proper information	3.27	3
